# A β–Sitosterol Encapsulated Biocompatible Alginate/Chitosan Polymer Nanocomposite for the Treatment of Breast Cancer

**DOI:** 10.3390/pharmaceutics14081711

**Published:** 2022-08-16

**Authors:** Obaid Afzal, Md Habban Akhter, Irfan Ahmad, Khursheed Muzammil, Adam Dawria, Mohammad Zeyaullah, Abdulmalik S. A. Altamimi, Habibullah Khalilullah, Shehla Nasar Mir Najib Ullah, Mohammad Akhlaquer Rahman, Abuzer Ali, Naiyer Shahzad, Mariusz Jaremko, Abdul-Hamid Emwas, Ibrahim Abdel Aziz Ibrahim

**Affiliations:** 1 Department of Pharmaceutical Chemistry, College of Pharmacy, Prince Sattam Bin Abdulaziz University, Al-Kharj 11942, Saudi Arabia; 2 School of Pharmaceutical and Population Health Informatics (SoPPHI), DIT University, Dehradun 248009, India; 3 Department of Clinical Laboratory Sciences, College of Applied Medical Sciences, King Khalid University, Abha 62521, Saudi Arabia; 4 Department of Public Health, College of Applied Medical Sciences, Khamis Mushait Campus, King Khalid University, Abha 62521, Saudi Arabia; 5 Department of Basic Medical Science, College of Applied Medical Sciences, Khamis Mushait Campus, King Khalid University, Abha 62521, Saudi Arabia; 6 Department of Pharmaceutical Chemistry and Pharmacognosy, Unaizah College of Pharmacy, Qassim University, Unaizah 51911, Saudi Arabia; 7 Department of Pharmacognosy, Faculty of Pharmacy, King Khalid University, Abha 62521, Saudi Arabia; 8 Department of Pharmaceutics and Industrial Pharmacy, College of Pharmacy, Taif University, Taif 21974, Saudi Arabia; 9 Department of Pharmacognosy, College of Pharmacy, Taif University, P.O. Box 11099, Taif 21944, Saudi Arabia; 10 Department of Pharmacology and Toxicology, Faculty of Medicine, Umm Al-Qura University, Makkah 21955, Saudi Arabia; 11 Smart-Health Initiative (SHI) and Red Sea Research Center (RSRC), Division of Biological and Environmental Sciences and Engineering (BESE), King Abdullah University of Science and Technology (KAUST), Thuwal 23955, Saudi Arabia; 12 Core Labs, King Abdullah University of Science and Technology (KAUST), Thuwal 23955, Saudi Arabia

**Keywords:** β–sitosterol, phytosterol, nanoparticle, breast cancer, chitosan, alginate

## Abstract

β–sitosterol is the most abundant type of phytosterol or plant sterol and can be found in various plant dietary sources including natural oils, soy products, and nuts. Numerous studies have demonstrated the potential therapeutic and clinical applications of β–sitosterol including lowering low-density lipoprotein and cholesterol levels, scavenging free radicals in the body, and interestingly, treating and preventing cancer. This study focuses on synthesizing and characterizing β–sitosterol encapsulated Alginate/Chitosan nanoparticles (β–sito–Alg/Ch/NPs) and evaluating their effectiveness in breast cancer treatment and their pharmacokinetic profile in vivo. The synthesized NPs, which incurred a mean size of 25 ± 1 nm, were extensively characterized in vitro for various parameters including surface charge and morphology. The NPs were further analyzed using DSC, FT-IR, thermogravimetry and X-ray diffraction studies. The release of β–sito from NPs was carried out in a bio-relevant medium of pH 7.4 and pH 5.5 and samples were drawn off and analyzed under time frames of 0, 8, 16, 32, 64, 48, 80, and 96 h, and the best kinetic release model was developed after fitting drug release data into different kinetic models. The metabolic activity of MCF-7 cells treated with the prepared formulation was assessed. The radical scavenging potential of β–sito–Alg/Ch/NPs was also studied. The pharmacokinetic parameters including C_max_, T_max_, half-life (t_1/2_), and bioavailability were measured for β–sito–Alg/Ch/NPs as compared to β–sito–suspension. The β–sito–Alg/Ch/NPs stability was assessed at biological pH 7.4. The % drug release in PBS of pH 7.4 reportedly has shown 41 ± 6% vs. 11 ± 1% from β–sito–Alg/Ch/NPs and β–sito–suspension. In acidic pH 5.5 mimicking the tumor microenvironment has shown 75 ± 9% vs. 12 ± 4% drug release from β–sito–Alg/Ch/NPs and β–sito–suspension. When compared to the β–sito–suspension, the β–sito–Alg/Ch/NPs demonstrated greater cytotoxicity (*p* < 0.05) and ~3.41-fold higher oral bioavailability. Interestingly, this work demonstrated that β–sito–Alg/Ch/NPs showed higher cytotoxicity due to improved bioavailability and antioxidant potential compared to the β–sito–suspension.

## 1. Introduction

Breast cancer has the highest incidence rate among women relative to other types of cancer [1]. As per current estimates, ~49,290 cases of ductal carcinoma and 101,280 cases of melanoma were diagnosed in the female breast in situ in 2021 [2]. In accordance with new cases, breast cancer alone accounts for 281,550 and constitutes 30% of all female cancers. There were 43,600 cases of death reported due to breast cancer alone in 2021. The death rate ranked among females retained 2nd spot due to breast cancer after lung cancer [2]. 

Conventional approaches for cancer treatment include chemotherapy, radiotherapy and surgical procedures. Although chemotherapy is the most common type of therapy, it is lacking in therapeutic efficacy due to incomplete eradication of the target disease, threatening toxicity, non-selective nature of drug delivery and serious adverse effects, in addition to multidrug resistance [3]. Moreover, the inadequate solubility and dissolution of common chemotherapeutics introduce further challenges to formulate them [4]. Thus, there is an urgent need for the development of a drug delivery system that can surmount the challenges in chemotherapy and provide safe and effective drug delivery to the target site while minimizing adverse effects to the neighboring cells or tissues.

A few decades back, the trends in developing dosage forms based on nanoparticulate systems achieved tremendous popularity and remain a viable therapeutic approach owing to the precise selectivity, specificity and targeting potential to curtail the off-target release and improve the drug concentration in the target domain [5]. Recently, tremendous improvements in the targeted drug delivery for cancer have been established by the application of a diverse range of nano-cargos such as polymer nanoparticles, e.g., chitosan, alginate, PLGA, PLA, micelles, as well as lipid nano-cargos such as liposome, ethosomes, glycerosome, and also inorganic particles such as silica NPs, magnetic NPs, dendrimers, quantum dots, carbon nanotubes, and fullerenes [6,7,8]. The leaky tumor vasculature, endothelial pores, and large fenestration lead to a disorganized microvasculature in the tumor microenvironment through which NPs could infiltrate leading to an enhanced permeation and retention (EPR) effect [9]. 

The passive mode of NP delivery to the tumor environment has the potential to enrich therapeutic concentrations via the EPR effect. However, it is important to make note of a few limitations that need to be addressed in the preclinical models. The growth of pressure in the interstitial fluid in tumor tissues and angiogenic factors which restrict the nano-cargos movement ultimately results in inadequate therapeutic drug delivery [10]. Chitosan forms a stable complex structure with alginate molecules via electrostatic attraction. The complex assists in the better entrapment of therapeutics and prevents drug loss through oxidation, hydrolysis and enzymatic degradation. The different functional groups present in chitosan molecules such as hydroxyls, carboxyls and amino groups serve as a target for chemical modification to improve solubility and dissolution, as well as improving the hydrophobic drug residence time within a nanosystem [11]. Alginate is a water-soluble polysaccharide made of α-L-guluronic and β-D-mannuronic acid residues naturally obtained from brown seaweed. The mucoadhesiveness, gelling property and compatibility with the biological components make alginate a promising carrier system for drug delivery and biomedical uses. Chitosan is a linear polysaccharide having a basic moiety of glucosamine and N-acetylglucosamine. It is widely exploited in drug delivery, biomedical and theranostic applications due to its biodegradable and biocompatible nature, possessing no toxicity in peroral drug delivery [12]. The aqueous dispersion of a complex of alginate and chitosan in calcium chloride encapsulating therapeutics formed through the ion gelation technique. The ionic gelation relies on interfacial polymerization between alginate and calcium chloride within the guluronic residue cavities resulting in the formation of ionic NPs [13].

The earlier work by Khalilullah and colleagues has shown the phytosterol-loaded surface-tailored bioactive Alginate/Chitosan NPs for treating breast cancer. They developed and characterized formulations in vitro for various parameters. The surface of the NPs was modified using conjugation chemistry for the active targeting of cancer. The surface-tuned NPs have shown a particle size of 126 ± 9 nm and % drug release was 72 ± 7% in the acidic pH [14]. The current study, however, reported a particle size of optimized formulation was 25 ± 1 nm and 75 ± 9% drug released in the acidic medium. Despite these, the current study also investigated the pharmacokinetic parameters and involved lesser steps in the manufacturing procedure. 

Since a few decades ago, interest in the therapeutic utility of natural products has grown dramatically due to their favorable properties when compared to synthetic or semi-synthetic drugs. It is commonly accepted that compounds from natural sources are well endured in the therapy of different ailments. For example, the implication of herbal moiety in various biological activities has been established so far including naringenin [15] sulphoraphane [16], plumbagin [17], α-mangostein [18] in anti-cancer therapy. Β–sitosterol, a plant sterol similar to cholesterol, is abundantly present in vegetable oils and food grains such as wheat, as well as in beans, corn, and dry fruits [19]. The potential lowering of cholesterol absorption in the small intestine warrants a wide range of beneficial biological activities of β–sitosterol, including cardiovascular protection by reducing low-density lipoprotein, preventing the formation of atherosclerotic plaques and suppressing cancer growth; these activities have been investigated in the literature [20,21,22,23]. Previous scientific studies reported that it has noteworthy biological effects such as anti-inflammation [24], antioxidant [25], anti-depressant [26], antibacterial [27], antihypertensive [28], anti-cancer [29], immunomodulator [30], anti-diabetic [25], anti-fungal [31], and wound healing properties [32]. 

## 2. Materials and Methods

### 2.1. Reagents

Sisco Research Laboratories Pvt. Ltd. Supplied Chitosan (Mw, 50–190 Kda, 75–85% deacetylation), β–sitosterol, and sodium alginate (Mw, 75–100 kDa). Qualigens fine chemicals (Mumbai, India) provided calcium chloride; Dimethyl sulphoxide (DMSO) was received from Merck, India. The reagents used in this study including HPLC grade water, and other solvents were analytical standards. The cancer cell line, MCF-7 was received from the National Centre for Cell Science (Pune, India). The cytotoxicity study was performed as per scientific literature. The suitable culture medium to keep cells growing and viable was modified Dulbecco’s medium with antibiotic, 100 mg/mL; Fetal Bovine Serum (10%); and penicillin (100 unit mL^−1^) in a controlled environment. The cell line was regularly monitored and incubated at 37 °C in a 5% CO_2_/95% controlled air environment. The buffering reagents used for the preparation of phosphate saline buffer (PBS) were standard analytical grade laboratory reagents received from a central drug house (New Delhi, India). Other laboratory reagents provided were employed as received.

### 2.2. Optimization

The 3 levels (−1, 0, +1), 3-factors, Box–Behnken design was used to optimize the formulation, Alg/Ch/NPs, as this design offers a more efficient and economic strategy. The formulation was optimized using Design-Expert software (Version 10; Stat Ease Inc., Minneapolis, MN, USA) software [15,16,33]. The list of independent variables with respective levels used in the study is shown in Table 1. The useful excipients selected under the study such as chitosan (X1), sodium alginate (X2), and calcium chloride (X3) as input attributes and their impact on various responses including particle size, (Y1), PDI (Y2) and entrapment efficiency, (Y3) were successfully studied in developing NPs has expressed in Table 2. The model analysis was ensured with a goodfit by adopting one-way ANOVA for developing an optimized formulation. The concluding optimum formulation of β–sito–Alg/Ch/NPs was brought forth by sustaining a measure of minimum particle size, PDI, and maximum entrapment efficiency, and the point-prediction technique was followed. For assessing the impact of variables on responses, statistical analysis was applied to investigate them. 

### 2.3. Alg/Ch/NPs Preparation

**Figure 1:**
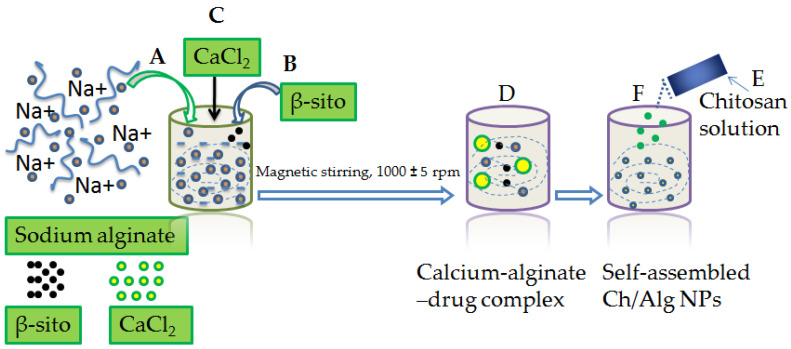
Diagram expressing steps in β–sito–Alg/Ch/NPs preparation. Aqueous alginate (1 mg/mL) solution pH lined up to 5.2 (**A**); Incorporation of β–sito solution to the alginate solution (**B**); Subsequent addition of CaCl_2_ solution to the alginate solution (**C**) via injectable needle measuring volume 0.5 mL/min following uninterrupted stirring, 1000 ± 5 rpm for 30 min, consequently entangling β–sito in a complex structure of calcium–alginate (**D**); Drop-wise addition of the chitosan solution to the alginate solution (**E**), a self-assembled β–sito–Alg/Ch/NPs formulation resulted (**F**).

The Alg/Ch/NPs bearing β–sito was prepared with the ion gelation technique such as the earlier reported method with modification [34,35]. Initially, a sodium alginate solution of 5 mL (1mg/mL) was prepared in distilled water and the solution pH was adjusted to 5.2 with 1M HCl. A total of 5 mg of β–sitosterol was separately dissolved in ethanol and sonicated, then injected drop-wise into the sodium alginate solution until the preparation turned uniform and homogeneous. Subsequently, 5 mL of calcium chloride was transferred to the sodium alginate solution slowly, 0.3 mL/min following uninterrupted magnetic stirring (1000 ± 5 rpm for 30 min) and a calcium chloride-alginate complex thereby formed encapsulating, β–sitosterol. A solution of chitosan was prepared by dissolving in acetic acid 1% (*v/v*) and 10 mL of this was injected slowly in a drop-wise manner into sodium alginate solution which led to the formation of auto-assembled NPs. Further, β–sito–Alg/Ch/NPs were magnetic stirred for 1 h to obtain uniform, homogeneity, and particles were disseminated further with probe sonication for 10 min (1 cycle, 100 W, 30 kHz power), and centrifuged for 15 min at 15,000× *g*. Moreover, NPs were made free of unloaded drug, free polymer and cross-linker using filtration, washing and lyophilized, thereafter, stored temporarily for characterization. The diagram showing the preparation steps involved in β–sito–Alg/Ch/NPs preparation is outlined in Figure 1.

### 2.4. Formulation Characterization

#### 2.4.1. Nanoparticle Size, Distribution Pattern, Zeta Potential and TEM

The particle size of the formulation was analyzed by Zetasizer and transmission electron microscopy (Techni TEM 200 Kv). The NP sample was diluted 10-fold in distilled water, and 10 μL of the diluted sample was disseminated on a carbon-layered copper grid, dried, then layered with phosphotungstic acid (1%) and analyzed at an induced voltage of 80–100 kV. Images of the NPs were examined. The zeta potential of β–sito–Alg/Ch/NPs was used to determine the surface charge of the NPs. It provides a key aspect of stability of the colloidal system.

#### 2.4.2. Entrapment Efficiency (EE) and Drug Loading (DL) 

The amount of drug encapsulated in the β–sito–Alg/Ch/NPs was measured by estimating the concentration of free drug in the supernatant. The drug loading capacity analyzed the drug encapsulated per unit weight of NPs. For measuring the *EE* and *DL*, 2 mL of NPs was centrifuged using a refrigerated centrifuge. The total quantity of supernatant was separated for estimating the free drug. The supernatant of 100 μL was extracted in a mixture of methanol:acetonitrile 30:70 (*v/v*) and the concentration of the drug was estimated with HPLC. The % *EE* and % *DL* were calculated applying the below-mentioned formula:

$$\%\;EE=\frac{\mathrm{Total}\;\mathrm{amount}\;\mathrm{of}\;\mathrm{drug}-\mathrm{Total}\;\mathrm{free}\;\mathrm{drug}\;\mathrm{in}\;\mathrm{the}\;\mathrm{supernatant}}{\mathrm{Total}\;\mathrm{amount}\;\mathrm{of}\;\beta –\mathrm{sito}}\times 100\;\%\;DL=\frac{\mathrm{Total}\;\mathrm{amount}\;\mathrm{of}\;\mathrm{drug}\;\mathrm{entrapped}\;\mathrm{in}\;\mathrm{NPs}}{\mathrm{Total}\;\mathrm{weight}\;\mathrm{of}\;\mathrm{NPs}\;}\times 100$$

#### 2.4.3. Differential Scanning Calorimetry

The melting point of β–sitosterol, chitosan, sodium alginate, and β–sito–Alg/Ch/NPs were performed using DSC apparatus. The two DSC pans were used for keeping the sample of ~5 mg in one pan and a reference standard on the other pan simultaneously. Both the pans keep on heating at a specific scanning rate to 350 °C applying a dry nitrogen gas.

#### 2.4.4. Thermogravimetric Analysis (TGA)

The study was executed using a thermogravimetric analyzer (Setaram, LABSYS EVO 1150 °C) for analyzing the thermal stability of β–sitosterol, excipients (chitosan, sodium alginate), and formulation, β–sito–Alg/Ch/NPs. The samples weighing 5 mg were subjected to heating at a temperature range of 0 °C to 350 °C with a maintained heating rate of 10 °C/min under an inert environment of nitrogen gas. The loss in sample weight was recorded with respect to the change in temperature.

#### 2.4.5. Fourier Transform Infrared Spectroscopy (FT-IR)

The FT-IR spectrometer (Bruker Corporation, Billerica, MA, USA) was used to analyze the pure β–sitosterol, chitosan, sodium alginate, and β–sito–Alg/Ch/NPs. The weight quantity (~5 mg) of individual components was kept in direct contact incident beam of light, and thus, the spectrum brought forth was set down in the array of 400–3500 cm^−1^. Therefore, the spectrum generated for excipients and formulation was set down and investigated.

#### 2.4.6. X-ray Diffraction Study

X-ray diffraction (X-rd) analysis of pure β–sitosterol, chitosan, sodium alginate, and β–sito–Alg/Ch/NPs were performed using simple phase analysis (Rigaku Ultima IV) operating at a voltage of 40 kV, and a current of 30 mA under monochromatic radiation of wavelength 1.5406 Å. The 2theta (2θ) angles were between (10 and 80°) with a scanning speed of 8°/min and a scintillation counter was employed as a detector.

#### 2.4.7. Drug Release Study

The percentage of drug dissolve from β–sito–Alg/Ch/NPs was comparatively evaluated in PBS of pH 7.4, and pH 5.5 with respect to β–sito–suspension. The dialysis membrane was activated in PBS solution prior to the offset of the dissolution study. The accurate weight quantity of each β–sito–Alg/Ch/NP, and β–sito–suspension having an equal dose of 10 mg were enclosed in a dialysis membrane previously filled with 100 mL of PBS with ends tightened, and dipped in the dissolution medium (37 ± 0.5 °C) and stirred continuously at 100 rpm [36]. The fixed volume of the sample (500 μL) was withdrawn at a different set of intervals (0, 8, 16, 32, 64, 48, 80, and 96 h), and the withdrawal volume was replaced with remaining PBS. The withdrawn analyte was estimated for drug quantity using HPLC. Moreover, the release kinetic model was implemented to the release data to screen out the best-fitted model by applying the graphical method to analyze the mechanism of drug release, and to finalize the best-suited model of good fit.

#### 2.4.8. Ex Vivo Drug Permeation Study

To study intestinal permeation, 180–200 g of rat from the control group was separated and their intestine was excised after sacrifice. The foreign particles such as cell debris and mucous over the excised part were properly cleaned using a PBS of pH 7.4. Formulations of β–sito–Alg/Ch/NPs having 5 mg of β–sitosterol were cautiously placed in the intestinal sac of which one end was ligated and the other end was also ligated after transferring the sample. Afterward, the intestinal sac was dipped in the receiver chamber bearing 100 mL, PBS and the organ was thoroughly aerated, and maintained the constant temperature of the receiver chamber at 37 ± 0.5 °C and keeps the samples agitated throughout the experiment. At a prefixed interval of time, 1 mL sample was withdrawn, and the same volume was replaced with fresh PBS. The quantification of the sample for the presence of the drug was performed using HPLC at 210 nm. Moreover, the quantity of β–sitosterol permeated from β–sito–Alg/Ch/NPs in comparison with β–sito–suspension inside the intestinal mucosa of rats at various time points and their flux were estimated.

#### 2.4.9. MTT Assay 

To investigate the cytotoxic effect of the developed dosage form, β–sito–Alg/Ch/NPs and β–sito–suspension were incubated with the MCF-7 cell line in increasing drug concentration. For this study, ~5 × 10^3^ cells were seeded into a 96-well plate and incubated at 37 °C for 24 h under humidified conditions supplied with 5% CO_2_. During the post incubation period, the culture medium was discarded, and each well was subjected to a varying concentration of β–sito–Alg/Ch/NPs (10–50 μM) and β–sito–suspension containing the same dose, and incubated subsequently for 24 h and 48 h at 37 °C. Then, the medium was superseded with an MTT reagent (0.5%, ~10µL) into an individual well, and incubated for 4 h. Moreover, the aliquot was bumped off and added 100 µL of dimethyl sulfoxide (DMSO) into each well. The absorbance was taken at a wavelength of 570 nm and recorded using a microplate reader (Bio-Rad, Hercules, CA, USA) [35]. The control group of cells remains untreated or in a blank preparation and is considered to be 100% viable cells. The doxorubicin was used as standard (positive control) due to a wider application against cancer therapy. The concentration at which 50% of cells remained viable was referred to IC_50_ and determined for β–sito–Alg/Ch/NPs and β–sito–suspension. The cell viability (%) was calculated as mean cell viability (%) ± standard deviation (SD) (*n* = 3) using the equation:

% Cell viability = Absorbance of sample/Absorbance of controlled × 100

#### 2.4.10. Pharmacokinetic Assessment

The animal studies were conducted in compliance with all the ethical research guidelines which were duly approved by the Institutional Ethics Committee, King Khalid University, Abha, Saudi Arabia (Approval No. ECM#2020–3219). A single-dose randomized study was applied for the assessment of pharmacokinetic parameters. The male albino rats from the control group (untreated) were categorized into two groups. The group I rats (*n* = 3) were given a single dose of formulation β–sito–Alg/Ch/NPs orally (20 mg/kg), and group II animals (*n* = 3) received a single dose of β–sito–suspension (20 mg/kg) orally. After administration of β–sito–Alg/Ch/NPs and β–sito–suspension, blood sample of 200 µL was withdrawn from tail vein in heparinized Eppendorf tubes at a fixed time interval (0, 0.5, 1, 2, 4, 6, 8, 10, and 12 h). Then, the samples were centrifuged at 4000 rpm for 20 min. The extracted plasma after centrifugation was kept at −20 °C until analysis. The plasma protein (50 µL) was treated with organic solvent dimethyl sulphoxide (DMSO), acetonitrile (250 µL) and followed by ethyl acetate (0.5 mL), sample vortexed for a few minutes and then centrifuged for 10 min at 12,000 rpm. The aliquot fraction of the sample was collected, and vacuum dried at 40 °C. Moreover, the residue was re-constituted in DMSO (100 μL), and 10 μL was injected in HPLC for analysis. The non-compartmental pharmacokinetic data analysis was used with PK solution 2.0 software to estimate pharmacokinetic parameters.

#### 2.4.11. Radical Scavenging Assay 

The radical scavenging power of both β–sito–Alg/Ch/NPs and β–sito-suspension were investigated by DPPH assay in accordance with the modified method reported in the literature [37]. The stock solution of β–sito–Alg/Ch/NPs and β–sito–suspension, each concentration of 10 mg/mL in DMSO was prepared. The different working standards from each stock solution were further prepared in the concentration range of (10–100 μg/mL). The freshly prepared 100 µL of DPPH reagent (0.02%) was mixed with each sample of volume 0.5 mL with vigorous shaking at room temperature and incubated for half an hour at 28 °C in dark conditions. After some time, the violet color solution changed to colorless owing to a reaction between the DPPH reagent and the sample having antioxidant potential. Ethanol of 50 μL was used in the preparation of the blank mixture. The decreased absorbance of the formulation and drug suspension was recorded at a wavelength of 517 nm using UV-Visible spectroscopy. The antioxidant capacity of β–sito–Alg/Ch/NPs, and β–sito–suspension was assessed in term terms of % inhibition in the DPPH assay. The reduced DPPH reagent concentration was measured from the sample calibration curve, and the assay was performed in triplicate (*n =* 3).

The % radical scavenging capacity was investigated using the equation:

*% radical scavenging capacity* = *Bo* − *B*1/*Bo* ×100

where *Bo* is the absorbance of blank; *B*1 absorbance of sample estimated from UV-Visible spectroscopy.

## 3. Formulation Stability 

The long-term stability of formulation β–sito–Alg/Ch/NPs in the colloidal state was measured with modification from the references [38,39,40]. The stability was determined at physiological pH of 7.4. The prepared sample, β–sito–Alg/Ch/NPs was kept in a stability chamber at a normal room temperature of 25 ± 2 °C, for three months. The β–sito–Alg/Ch/NPs were supervised at intervals of 0, 30, 60, and 90 days for changes acquired in particle size, PDI, zeta potential, % entrapment efficiency, and % drug loading. To validate the reproducibility of obtained results, the measurements were carried out in triplicate (*n* = 3).

## 4. Data Analysis

The statistical analysis was accomplished using one-way ANOVA accompanied by Tukey–Kramer analysis applying version 7 GraphPad prism. The data gathered were represented by average ± standard deviation (*n* = 3). The statistical significance is considered when, (*p* < 0.05).

## 5. Results and Discussion

### 5.1. Optimum Formulation

**Figure 2:**
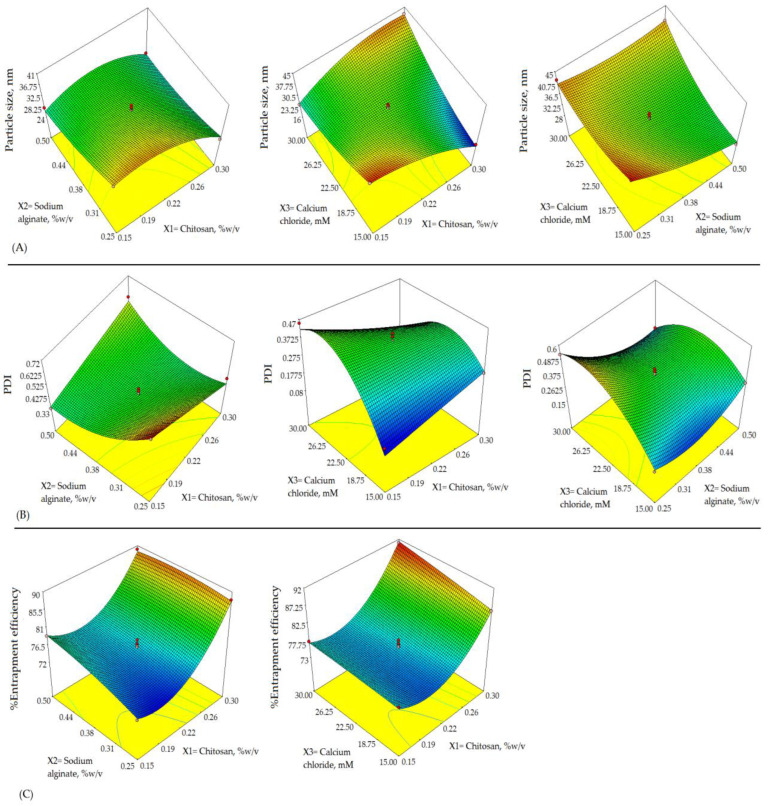
Response surface morphology in three-dimensional (3D) plot (**A**–**C**) exemplifying the effects of independent variables, X1: Chitosan, %*w*/*v*; X2: Sodium alginate, %*w*/*v*; and X3: Calcium chloride, mM on responses, Y1: Particle size, nm; Y2: PDI; and Y3: Entrapment efficiency, %.

The alginate/chitosan nanoparticulate preparation was optimized by applying 3^3^-Box Behnken Design within a specific time. The chosen formulation variables viz., X1, X2, and X3, applied in 3-levels, low (−1), medium (0), and high (+1) were contemplated in the responses such as Y1, Y3, and Y3, respectively. The levels for the variables used have shown in Table 1. These levels (−1, 0, +1) were selected based on a preliminary examination. Two-dimensional (2D) counterplots and 3D response surface plots defined the impact on responses of different formulations as depicted in Figure 1 and Figure 2. The experimental ranges of independent variables selected as low (−1) and high (+1) were based on the investigation at the preliminary stage of the drug development process and published data of folate armoured alginate/chitosan NPs and within these ranges, the most stable and robust formulation could be ensured [14]. The best-fitted model was quadratic as indicated by the coefficient of correlation (R2)~1 in determining the effect of formulation variables on the responses. 

The quadratic equation was established based on the best-fitted model which explicated the possible interactions such as, individual, combined and quadratic effects on the responses. The statistical design has shown seventeen formulations, accompanied by 5-center points to study any misplay in the five copied preparations, as indicated in Table 2. The regression analysis results of responses Y1, Y2, and Y3 for data fitting into various models viz., quadratic, linear, 2FI, and cubic are shown in Table 3. The model summary statistics results determined the best-fitted model was quadratic as shown in Table 3.

### 5.2. Impact of Formulation Variables on Y1

The below-mentioned equation details the impact of formulation variables, on Y1 as follows;

Particle size = +33.30 + 8.00 × A − 3.12 × B + 1.13 × C − 2.00 × A × C + 2.25 × B × C − 7.53 × A^2^ + 1.22 × B^2^ + 6.73 × C^2^

**Figure 3:**
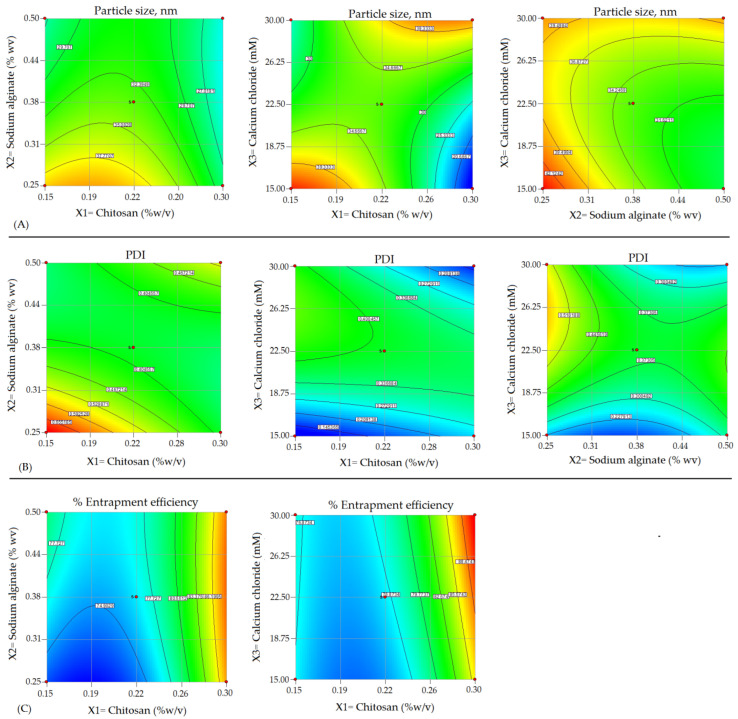
Two-dimensional contour plots (**A**–**C**) exhibiting the effect of X1: Chitosan, %*w*/*v*; X2: Sodium alginate, %*w*/*v*; and X3: Calcium chloride, mM on responses, Y1: Particle size, nm; Y2: PDI; and Y3: Entrapment efficiency, %.

The contour plot (Figure 3), 3D plots (Figure 2) and above quadratic equation of the particle size revealed a considerable negative effect of chitosan. The particle sizes of β–sito–Alg/Ch/NPs obtained for various formulations as shown in Table 2 ranged between 16 and 45 nm. The size distribution pattern of chitosan/alginate NPs was observed as narrow and consistent. At a polymer concentration of 0.15% *w/v*, the minimum particle size achieved 16 nm in the case of formulation Run 11, similarly, at the same polymer concentration, the particle size achieved 33.00 nm, 33.60 nm in case of Run 13 and 14, respectively. As per the quadratic equation of the particle size, the cross-linker had a positive impact on particle size. The higher concentration of cross-linker may accelerate the calcium alginate gel formation and thereby prompt complex formation with cationic chitosan and stabilize the nanocarrier system. Adversely, the higher concentration of alginate and calcium ions (Ca^2+^ ion) may advocate significant interaction of the carboxylic group of alginate with Ca^2+^ ions resulting in increased particle size [41,42]. Despite these, molecular weight, the degree of deacetylation of chitosan, and the ratio of alginate/chitosan had an impact on particle size [43]. 

A further increase in Ca^2+^ ion concentration to 0.30% *w/v* increased the entrapment efficiency as observed in formulation Run 3 to 91%. At a higher concentration of Ca^2+^ ion, entrapment efficiency increased which could be due to particle size reduction leading to a higher surface area. The increased surface creates an opportunity to increase the drug encapsulation due to the availability of more spaces. 

### 5.3. Impact of Formulation Variables on Y2

The impact of independent variables on the polydispersity index is shown in the quadratic equation below;

PDI = +0.38 + 0.17 × A − 0.026 × B + 0.082 × C − 0.58 × A × B − 0.033 × A × C − 0.082 × B × C + 0.076 × A^2^ − 0.058 × C^2^

The above equation for PDI, 3D-surface plot (Figure 2) and contour plot (Figure 3) has expressed an important effect on PDI. Both chitosan and alginate had a slightly negative impact on PDI. As per literature, the PDI value represents the NPs size distribution pattern in the bulk of formulation, the preparation with a larger particle size has a higher PDI value and vice-versa. Further, the low value of PDI indicates a monodisperse, homogeneous and stable system, and a high PDI value leading to wider distribution of particle size may be due to the formation of aggregates, floccules resulting in poor stability of the nanosystem. The chitosan concentration had a less positive impact on PDI, raising chitosan quantity may raise the viscosity of the nanosystem leading to the aggregation of particles via binding to the surface matrix resulting in larger particles and high PDI [44]. Alginate concentration had less of a negative effect on PDI. A low alginate concentration minimizes the electrostatic attraction with the cross-linker, Ca^2+^ ion leading to increased particle size, decreases the nanoparticle homogeneity, and thus increases PDI.

### 5.4. Impact of Formulation Variables on Y3

The polynomial equation showing the influence of independent variables on % entrapment efficiency of chitosan NPs is given below;

Entrapment efficiency = + 76.46 + 5.93 × A + 1.62 × B + 1.45 × C − 1.2 5 × A × B + 1.10 × A × C − 0.50 × B × C + 6.60 × A^2^ − 0.80 × B^2^ − 0.16 × C^2^

The entrapment efficiency of in-house built β–sito–Alg/Ch/NPs was calculated in the range of 72 to 91%. The effect on % drug encapsulation owing to the formulation variables according to the above quadratic equation of % entrapment efficiency, 3D response-surface curve (Figure 3) and contour plot (Figure 3) had explicated a blended impact. 

The above polynomial equation indicates that chitosan had a positive impact on % drug entrapment. At a low chitosan concentration of 0.15% *w/v*, the observed value of entrapment efficiency was 73% as indicated in Run 10. At a chitosan concentration of 0.30% *w/v*, the maximum observed entrapment efficiency was 91% in the case of formulation Run 3. Thus, herein the observation led to increasing chitosan concentration and increased the % entrapment efficiency of the drug. In agreement with our results, Xu associates reported similar outcomes from chitosan nanoparticles of protein, BSA. The reported study observed a negative impact on % entrapment efficiency of chitosan concentration probably due to enhanced viscosity and formation of aggregates or high consistency may resist drug entrapment of free drug in the bulk of solution [45]. The current optimized chitosan/alginate preparation at the presented chitosan concentration produced homogeneous and consistent results. Similarly, the gel-forming capability of alginate may impart a positive impact on entrapment efficiency that provided high drug holding capacity. The individual effect of the Ca^2+^ ion at low concentrations increased the entrapment efficiency of the chitosan NPs to 91% as shown in formulation Run 3, whereas, at the higher concentration it was decreased. However, the combined impact of the cross-linker Ca^2+^ ion with reverse-charged chitosan molecule led to improved drug entrapment may be attributed to the complex alginate/chitosan NPs. Oppositely, the combined effect of chitosan and alginate reduced the entrapment capacity of the drug, this could be ascribed to the competition of the binding site of chitosan and drug onto the alginate 1, 4-glycosidic linkages moiety [46].

### 5.5. Validation and Optimum Checkpoint Analysis 

The validation of the design of the experiment was achieved by investigating a few experiments under optimum conditions and finding agreement in experimental outcomes with respect to the predicted value. The numerical optimization solution tool was used to finalize the optimum preparation considering criteria of minimum particle size, PDI and maximum % entrapment efficiency. The desirability of optimum formulation was reported to be 0.851; explicating the robustness and consistency of the developed formulation. Post validation, the optimum concentration of the developed β–sito–Alg/Ch/NPs was chitosan 0.15% *w*/*v*; sodium alginate, 0.50% *w/v*; and calcium chloride, 15.07 mM. The predicted value of dependent variables were; particle size (17.14 nm), PDI (0.240), % entrapment efficiency (79.19%) and the experimental value of particle size, PDI and % entrapment efficiency were 25 ± 1 nm, 0.231 and 86 ± 3%, respectively. Further, the loading efficiency of the drug in optimized preparation was determined to be 8 ± 2%. The optimum formulation concentration of in-house build β–sito–Alg/Ch/NPs of observed vs predicted response with percentage error (Y1 = 4.7%; Y2 = 3.8%; Y3 = 8.7%) in Box-Behnken design are indicated in Table 4. The changes in the experimental value in contrast to the predicted one were found to be statistically insignificant (*p* < 0.05). Moreover, the zeta potential of the NPs was estimated +24 ± 4 mV, due to the surface positive of chitosan which explicated the stable nature of the optimized formulation. Further, homogeneity and uniform particle distribution of the optimum formulation was indicated by a low value of PDI [47].

### 5.6. Physico-Chemical Characterization of Formulation

#### 5.6.1. % Entrapment and Loading Efficiency, Particle Size, PDI, Zeta Potential, and TEM

**Figure 4:**
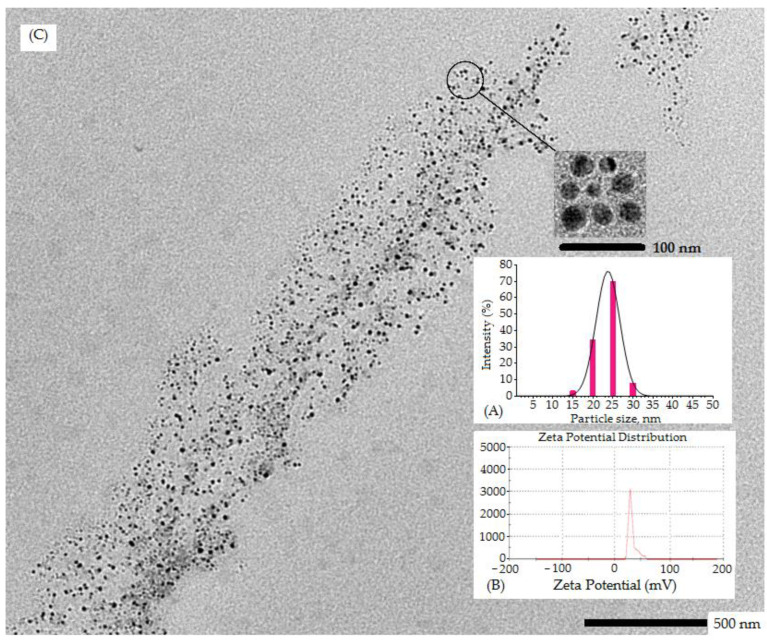
Particle size distribution indicated by red bar (**A**); Zeta potential (mV) (**B**); and transmission electron microscopic image of optimized β–sito–Alg/Ch/NPs (**C**).

The optimum particle size, zeta potential and PDI of developed β–sito–Alg/Ch/NPs were determined 25 ± 1 nm, +24 ± 4 mV, and 0.231. The entrapment and loading efficiency were determined at 86 ± 3%, and 8 ± 2%, respectively. The uniform, consistent and unimodal distribution of particle population and sizes that were less than 50 nm was confirmed in the formulation. The low value of PDI (0.231) indicated a homogeneous and monodispersed nanosize system. The zeta potential onto the surface of β–sito–Alg/Ch/NPs was noted +24 mV, indicating a positive surface charge bearing chitosan NPs. This could be due to the protonated amino group in chitosan that led to colloidal stability to the NPs and impersonated significant prediction of the fate of NPs in vivo [43]. The particle size distribution curve and zeta potential of β–sito–Alg/Ch/NPs have shown in Figure 4. As expressed in Figure 4, the TEM images of NPs were nanosize, spherical, uniform, non-aggregated and consistent which was well supported by the investigation executed by Malvern Zetasizer.

#### 5.6.2. Thermal Analysis

##### Differential Scanning Calorimetry

**Figure 5:**
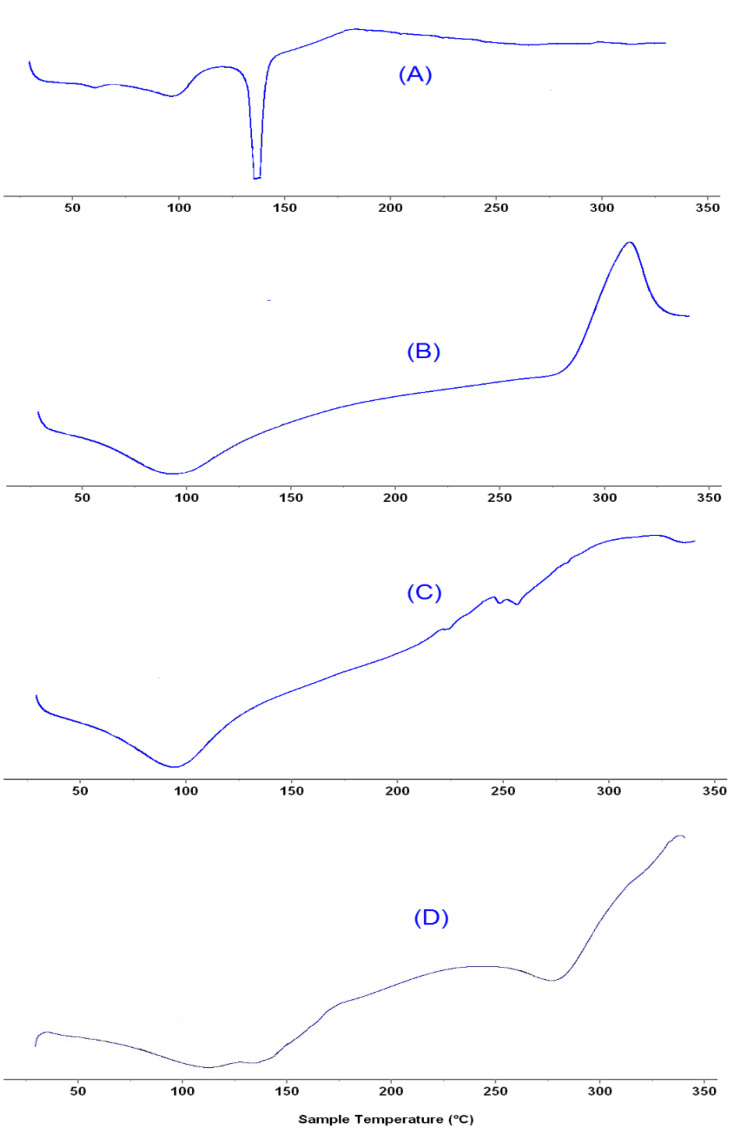
DSC thermogram expressing melting-point of β–sitosterol (**A**); Chitosan (**B**); sodium alginate (**C**); and β–sito–Alg/Ch/NPs (**D**).

The DSC thermogram of pure β–sitosterol, sodium alginate, chitosan and β–sito–Alg/Ch/NPs are depicted in Figure 5A–D. The pure β–sitosterol has shown an endothermic peak which appeared at a melting point of 137.89 °C, expressing their crystalline state (Figure 5A). The thermogram of chitosan appeared at a melting point of 93.36 °C, and 311.98 °C (Figure 5B), and sodium alginate at 93.82 °C (Figure 5C), respectively. Furthermore, the formulation of the drug with sodium alginate and chitosan underwent a complex formation and ensuing reduced crystalline structure of the drug in the formulation, indicated by peak flattening (Figure 5D) as well as lowering of the melting point of the drug [46].

##### Thermogravimetric Analysis (TGA)

**Figure 6:**
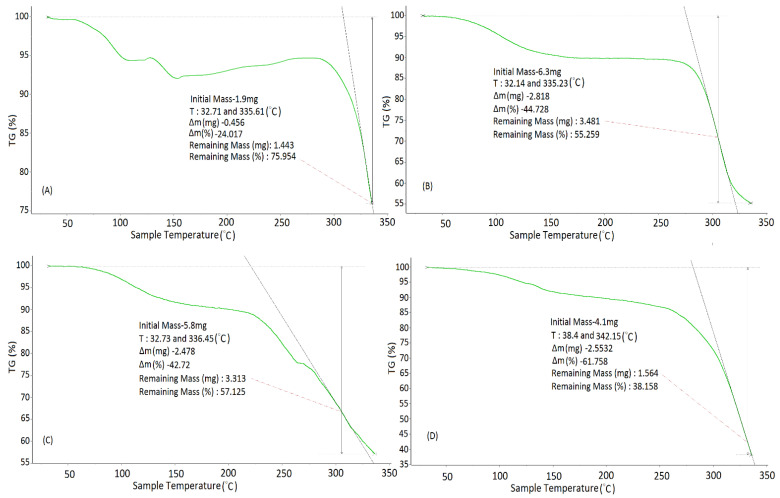
TGA analysis of β–sitosterol (**A**); Chitosan (**B**); Sodium alginate (**C**); and β–sito–Alg/Ch/NPs (**D**). Green line (**A**–**D**) indicates the % mass loss in samples.

This study was performed to investigate the thermal stability of pure polymers, chitosan and sodium alginate used in the formulation, as indicated in Figure 6A–D. A weight loss of 24% was perceived in the case of β–sito after approaching the temperature of 335.61 °C (Figure 6A). A 44.72% weight reduction in chitosan was observed as the temperature increased to about 335.23 °C (Figure 6B). Similarly, a loss of weight of 42.72% was observed in sodium alginate when the temperature approached 336.45 °C (Figure 6C). In the case of β–sito–Alg/Ch/NPs, the weight loss of 61.45% was perceived when the temperature reached 342.16 °C and it was due to the combined decomposition of β–sito, chitosan, and sodium alginate. The higher weight loss in the formulation intimidating that β–sitosterol is encapsulated in the polymeric matrix. The increased weight loss in the formulation of chitosan/alginate NPs of doxorubicin was also reported in the preceding work [35]. Further, chitosan and alginate weight loss due to degradation and carbonization at a temperature range of 200–360 °C was reported in the literature [48]. The degradation in the polymer is due to depolymarization, saccharide unit dehydration and the degradation of the polymers.

#### 5.6.3. FT-IR Spectral Analysis

**Figure 7:**
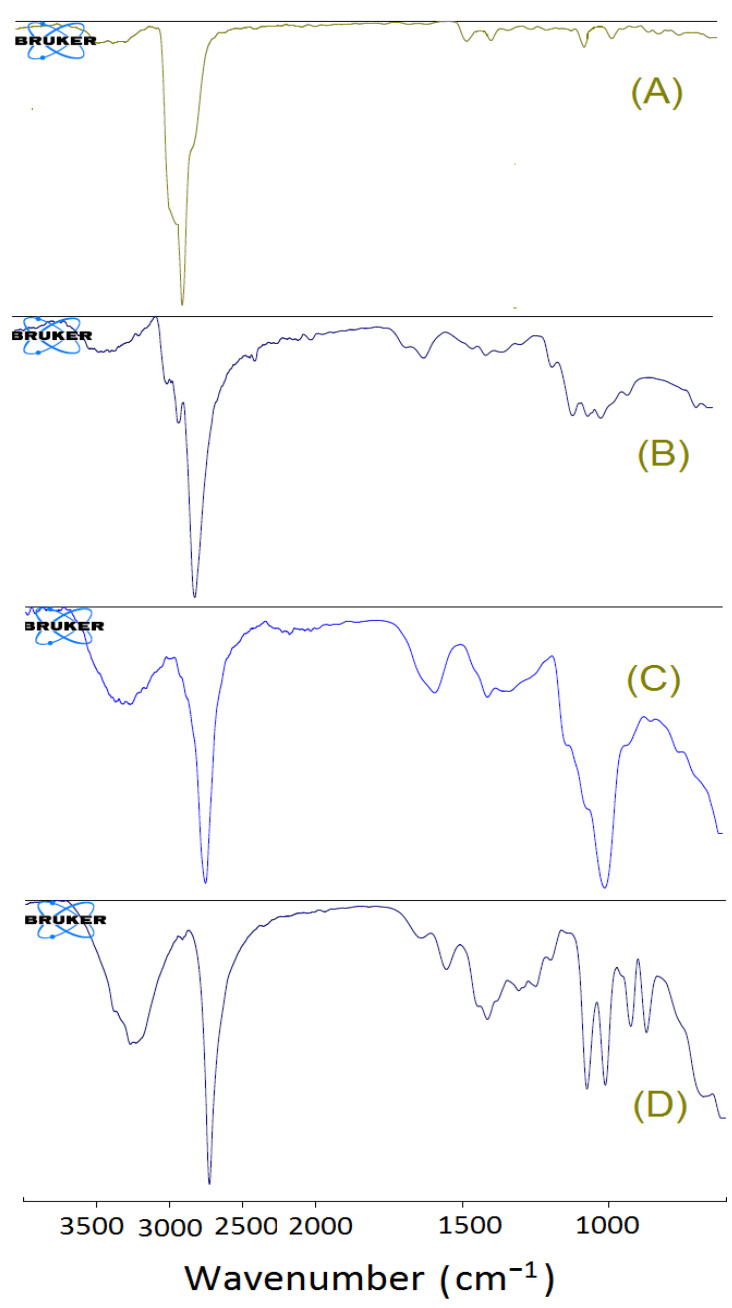
FT-IR spectra of β–sitosterol (**A**); Chitosan (**B**); Sodium alginate (**C**); and β–sito–Alg/Ch/NPs (**D**).

The interaction among the excipients under study, i.e., physiochemical interaction and chemical stability of the formulation were assessed by FT-IR spectroscopic analysis. The spectra of pure β–sito, chitosan, sodium alginate, and β–sito–Alg/Ch/NPs are showcased in Figure 7A–D. The chitosan showed characteristic peaks at 3359.22 cm^−1^ due to -NH2 and O-H stretch, at 2927.92 cm^−1^ due to C-H stretching, at 2838.25 cm^−1^ due to -C-H stretch. A sharp and strong peak was found at 2736.18 cm^−1^ due to -CH stretch, at 1647.49 cm^−1^ (C=O stretching), at 1588.05 cm^−1^ (-CONH2), at 1375.44 cm^−1^ (OH bending), at 1149.10 cm^−1^ (C-N stretch), at 1079.13 cm^−1^ (C-O stretching), and at 893.01 cm^−1^ stretching vibration due to saccharide ring. The alginate showed a characteristic peak at 3259.9 cm^−1^ due to -NH2 and O-H stretch, at 2745.36 cm^−1^ (-CH stretch), at 1591.31 cm^−1^ (N-H stretch), at 1410.67 cm^−1^ (carboxylate group), and at 1010.78 cm^−1^ (C-N stretch), respectively. The -NH2 and O-H group stretch in chitosan at 3359.22 cm^−1^ shifted to 3273.57 cm^−1^ in β–sito–Alg/Ch/NPs may be due to binding with alginate following complexation. The interpretation of the study disclosed that the carboxyl group in alginate binds with an amine group of protonated chitosan. The minor shift in the NPs expressed a complex formation in the polymeric matrix with the drug. Thence, the IR spectra of various components in formulation substantiated the chemical stability of the drug. The results noticed were consistent with the preceding work [35].

#### 5.6.4. X-ray Diffraction Study

**Figure 8:**
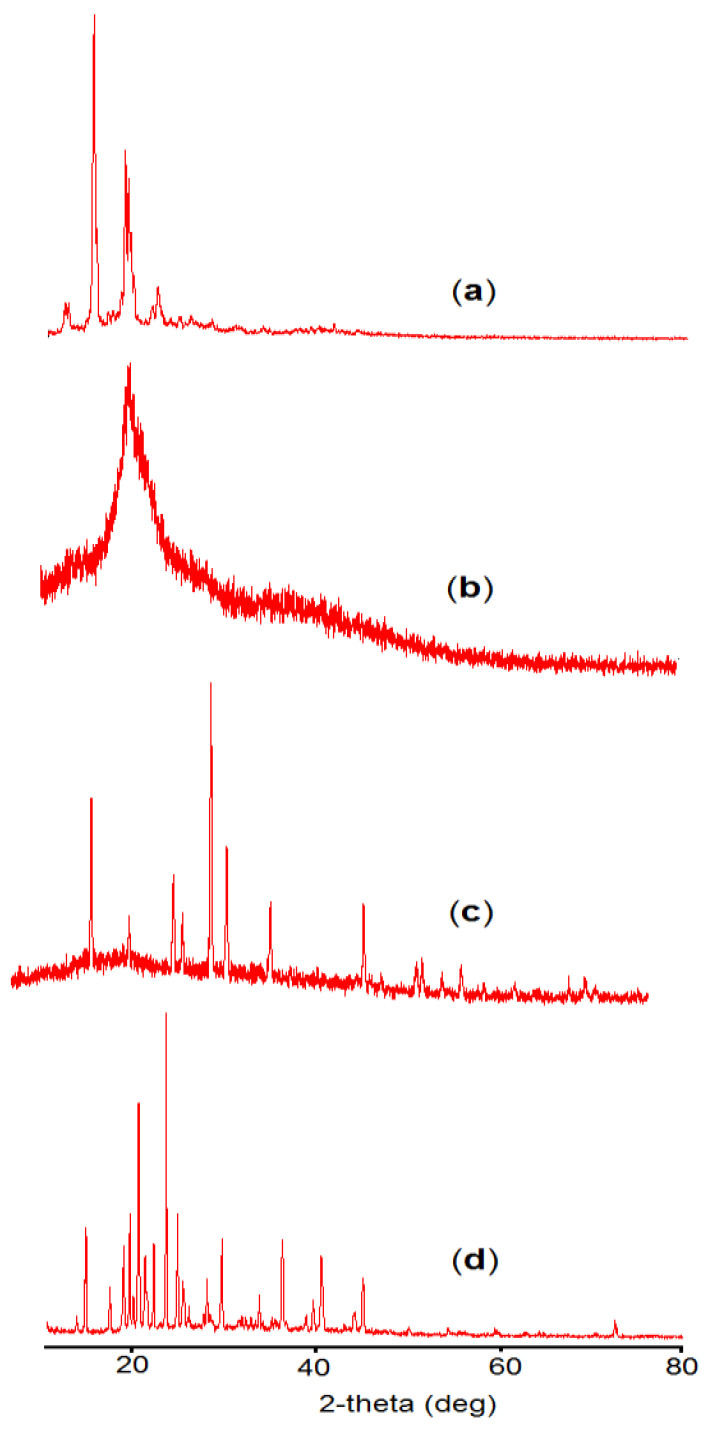
X–rd study of β–sitosterol (**a**); Chitosan (**b**); Sodium alginate (**c**); and β–sito–Alg/Ch/NPs (**d**).

X-ray analysis of β–sitosterol, chitosan, sodium alginate, and β–sito–Alg/Ch/NPs were performed as expressed in (Figure 8a–d). The crystalline structure of the compound is indicated by the presence of a sharp and intense peak (Figure 8a). Chitosan is characterized by the absence of an intense peak, a wide peak observed at a 2θ angle of 19.74° and the physical state was amorphous (Figure 8b). On the other hand, sodium alginate had shown some sharp peaks at 2θ angles of 19.039°, 23.2°, 33.844°, 48.758°, and 72.87° indicating some degree of crystallinity as expressed in (Figure 8c). The X-rd pattern in the formulation, β–sito–Alg/Ch/NPs showed some intense peaks which were plausibly from the sodium alginate at 2θ angles of 19.346°, 23.260°, 33.492°, and 72.324° and from chitosan at 2θ angle of 19.71°. However, the crystalline peak of the drug disappeared in the formulation because the drug remains inside the polymeric core in a molecular state [49].

#### 5.6.5. Release Studies and Kinetic Model

**Figure 9:**
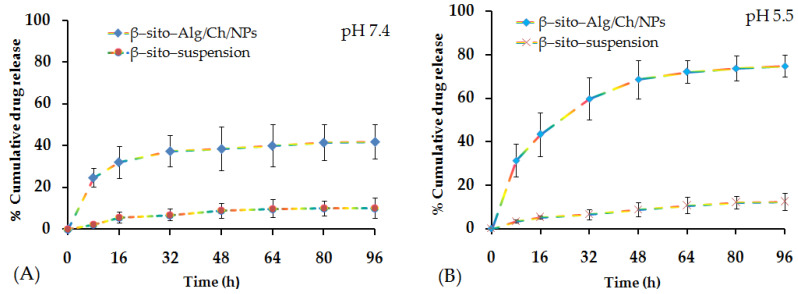
% Cumulative drug release from β–sito–Alg/Ch/NPs in comparison with β–sito–suspension in pH 7.4 (**A**); pH 5.5 (**B**); the sampling interval was 0, 8, 16, 32, 64, 48, 80, and 96 h, respectively.

The cumulative drug release profile at various time intervals has been estimated from β–sito–Alg/Ch/NPs relative to β–sito–suspension as shown in Figure 9A,B. The rapid drug release was observed in the initial phase of 8 h, i.e., 25 ± 5% and 31 ± 5% in pH 7.4 and pH 5.5 from β–sito–Alg/Ch/NPs. This burst release could be due to weakly held drug particles on the nanocarrier surface or improved solubilization or drug excess near the particle surface. Thereafter, it experienced a maximum release of 38 ± 11% (pH 7.4), and 69 ± 9% (pH 5.5) till 48 h from β–sito–Alg/Ch/NPs. The drug release rate was steady between 48 h and 96 h. The drug release from β–sito–suspension in PBS of pH 7.4 was 5 ± 1%. The release rate from drug suspension was slow and low due to the hydrophobic nature of the drug resulting in poor dissolution and release. However, drug release from β–sito–Alg/Ch/NPs and β–sito–suspension reportedly has shown 41 ± 6% and 11 ± 1% in pH 7.4. On the contrary, the drug release at an acidic pH of 5.5 simulating the tumor microenvironment from β–sito–Alg/Ch/NPs was 75 ± 9% compared to 12 ± 4% release from β–sito–suspension in the same pH. The drug release from β–sito–Alg/Ch/NPs was significantly higher than β–sito–suspension in either pH 7.4 or pH 5.5 (*p* < 0.05). In particular, the drug release from β–sito–Alg/Ch/NPs in pH 5.5 was noted as higher than pH 7.4. The higher drug release ascertained in the acidic medium may be attributed to the annihilation of the bond between the drug and Alg/Ch complex resulting in prompt dissolution and drug release from the polymeric matrix. It is worthwhile to note that the acidic tumor microenvironment favors swelling of chitosan and reduced electrostatic interaction, which together lead to higher drug release. The release profile observed herein is in agreement with preceding studies reported in the literature [35,50]. Further, the swelling of chitosan in an acidic medium is also supported by the repulsive forces that exist between the positively charged chitosan on the alginate complex with positively charged calcium ion, which sparks off the dissociation of the complex resulting in improved drug release in pH 5.5 than pH 7.4. In addition, the mechanism of drug release from alginate/chitosan complex depended on the degree of hydration in the aqueous medium of polymer matrix followed by gel swelling, resulting in the erosion of gel-polymer and drug diffused through them in the medium. The early fast drug release from nanocarrier may be ascribed to the alteration in the particle surface area and size due to the surface dissolution of alginate resulting reduce particle diameter [51]. Sodium alginate concentration in the formulation plays an important role in drug release rate, as it was reported that higher concentration led to sustained release over an extended time. Additionally, drug release from the Alg/Ch polymer matrix depends upon the pH of the surrounding medium, molecular state of the drug, drug dose, and other physicochemical features of the nanocarrier system [52].

The drug release data were fitted to different kinetic release models to predict drug release mechanism from polymeric carrier system, viz., zero-order, first-order, Higuchi, Korsmeyer–Peppas, and Hixson–Crowell, and to delineate the model with good data fit. The release kinetics model was selected based on the highest value of the coefficient of correlation (R^2^) from various models. It was seen that after fitting the data, Korsmeyer–Peppas, the best-fitted model, with (R^2^ = 0.9715) was selected. The exponent (*n*) value, calculated was 0.389 (0.5 < *n* < 1), intimating the Fickian diffusion mechanism for drug-release from β–sito–Alg/Ch/NPs [34]. The controlled drug release from NPs was governed by an aqueous incursion of water in the polymer matrix, hydration, swelling and matrix erosion, and thus diffusion of drug in the surrounding medium. Furthermore, the drug release is also influenced by the physiochemical property of the drug, pH of the medium, dose, and the polymeric layer of encapsulated drug [34].

#### 5.6.6. Intestinal Permeation Study 

**Figure 10:**
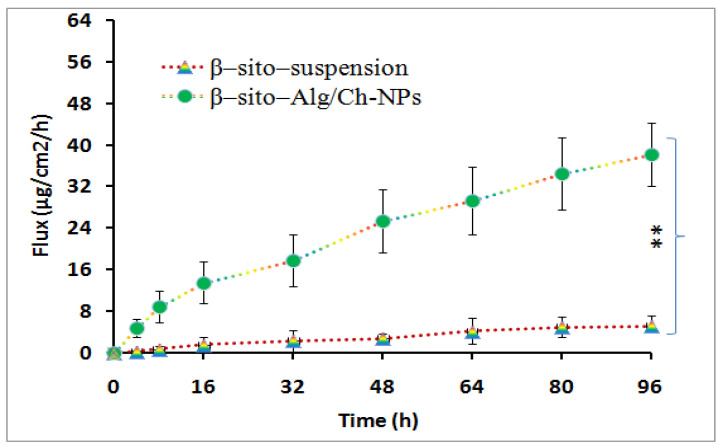
The ex vivo intestinal permeation experiment expresses flux of β–sito from β–sito–Alg/Ch/NPs and β–sito–suspension. Data expressed as mean ± SD in triplicate (*n* = 3) level of significance (** *p* ≤ 0.01).

The result of the intestinal permeation study has been outlined in Figure 10, which revealed a significant amount of β–sito has permeated from the optimized formulation in the intestinal mucosa as compared to β–sito–suspension. The maximum flux of β–sito determined from β–sito–Alg/Ch/NPs was 38 ± 7 vs. 5 ± 2 μg/cm^2^/h from β–sito–suspension. The flux of β–sito permeated across intestinal mucosa was significantly higher from β–sito–Alg/Ch/NPs than β–sito–suspension (*p* < 0.01). The protonated amino group on the surface of chitosan creates a positive charge which favors transport across the negative charge surface of the mucous membrane due to nano-biointeraction, thereby opening the tight junction of the intestinal cells. This brings to higher quantity of drug transport due to higher permeation via the mucosal layer of the intestinal membrane [53].

#### 5.6.7. MTT Assay

**Figure 11:**
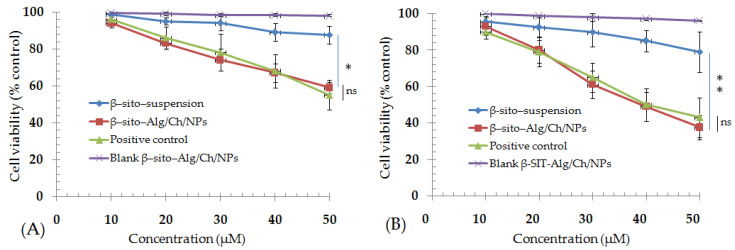
Cell viability (% control) after treatment with β–sito–Alg/Ch/NPs and β–sito–suspension at a dose ranges 10–50 µM effectively regressed the breast cancer cell line after an incubation time of 24 h (**A**) and 48 h (**B**). Data expressed as mean ± SD in triplicate (*n* = 3). Results estimated statistically using one way ANOVA and Tukey’s multiple comparisons test (* *p* < 0.05), (** *p* < 0.01), ns-not significant (*p* > 0.05) when β–sito–Alg/Ch/NPs compared with β–sito–suspension.

The cytotoxicity measurement using MTT assay was based on the formation of formazan crystals, (purple color) from yellow-color MTT dye which is being reduced by the viable cells. The decrease in cell viability following treatment with different concentrations of the formulation was ascertained as concentration and time dependent. The cell viability was reduced to 59 ± 3% and 88 ± 5% after a 24 h of formulation treatment by β–sito–Alg/Ch/NPs and β–sito-suspension. Similarly, in the next course of treatment for 48 h, the % cell viability was measured at 38 ± 7% and 79 ± 11% corresponding to β–sito–Alg/Ch/NPs and β–sito-suspension (Figure 11). The % cell viability shown by β–sito–Alg/Ch/NPs was statistically not significant as compared to positive control in either 24 h or 48 h of treatment (*p* > 0.05). The % cell viability exerted by standard Dox solution was comparable with the reported literature [54]. The IC_50_ of formulation β–sito–Alg/Ch/NPs and β–sito–suspension were 60 ± 4 and 181 ± 11 μM after completion of 24 h. Moreover, the IC_50_ was calculated accompanying 48 h of cell line incubation and the resulting concentration was determined to be 40 ± 3 μM and 121 ± 8 μM, equating to β–sito–Alg/Ch/NPs and β–sito-suspension. The finding suggested that the β–sito–Alg/Ch/NPs was significantly (*p* < 0.01) cytotoxic against breast cancer cells as compared to β–sito–suspension.

The passively targeted drug-loaded nanocarriers render the drug to the target domain or neighboring to the target cells/tissues thereby enriching the therapeutic efficacy. The transportation of the nanocarrier to the target increases the drug concentration and steadily increases the permeation of the drug to the affected cells/tissue. Although, the EPR-based targeting approach relies on the tumor type, architect of the tumor microenvironment and the animal xenograft model [55,56]. The NPs provided protective shielding to the phytoactive/therapeutics against degradation, making the system robust and stable. The content released by the nanocarrier is transported through passive targeting to the cells/tissue in the physiological fluid and integrated with biological constituents viz., plasma protein in the blood, thus enhancing the anti-proliferative effect against the cancer cells [57]. Oppositely, the poor death of cells observed from β–sito–suspension may be due to hydrophobic, unformulated features, and thus, limited their biological activity.

#### 5.6.8. Pharmacokinetic Assessment

**Figure 12:**
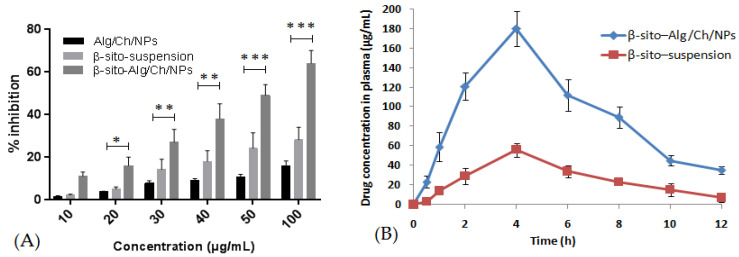
The comparative radical scavenging activity by DPPH assay of β–sito–Alg/Ch/NPs,β–sito–suspension and control (Alg/Ch/NPs) (**A**). Statistical significance “*” indicates significant * *p* < 0.05, “**” highly significant ** *p* < 0.01 value, and “***” extremely significant *** *p* < 0.001 when compared with β–sito–suspension. Comparative pharmacokinetic profile of β–sito–Alg/Ch/NPs and β–sito–suspension (**B**). Data expressed as mean ± SD in triplicate (*n* = 3).

The pharmacokinetic profile of β–sito–Alg/Ch/NPs was analyzed in comparison to β–sito–suspension (Figure 12B) and the data obtained are shown in Table 5. The peak plasma concentration (C_max_), and area under curve (AUC)_0−t_ determined for formulation, β–sito–Alg/Ch/NPs were 180 ± 0.02 μg/mL; 1080 ± 1 μg·h/mL, respectively. Adversely, the C_max_ and (AUC)_0−t_ determined for β–sito–suspension were 56 ± 0.2 μg/mL; 317 ± 1 μg h/mL, respectively. The results indicated improved absorption of β–sitosterol from formulation β–sito–Alg/Ch/NPs, as compared to β–sito–suspension (*p* < 0.01). The β–sito–Alg/Ch/NPs formulation demonstrated ~3.41-fold higher oral bioavailability than β–sito–suspension. The t_1/2_ of β–sito–Alg/Ch/NPs had increased to 5 h from 2 h measured for drug suspension. The maximum time to achieve plasma concentration (T_max_) achieved the same for formulation and drug suspension, i.e., 4 h. The elimination rate constant (K_e_) for β–sito–Alg/Ch/NPs and β–sito–suspension was measured at 0.2 ± 0.01 and 0.3 ± 0.02 h^−1^, respectively.

#### 5.6.9. Radical Scavenging Assay

The antioxidant capacity via DPPH assay is widely explored for the determination of free radical scavenging activity of the phytoactives and other compounds [36]. In this assay, the DPPH acts as a free radical which accepts protons (H^+^) from the compound (proton donor) whose antioxidant activity is to be measured. The experiment on this assay revealed that the β–sitosterol in the formulation has shown concentration-dependent radical scavenging properties. The comparative antioxidant activity of β–sito–Alg/Ch/NPs, β–sito–suspension and Alg/Ch/NPs (control) is expressed in Figure 12. The antioxidant capacity of the β–sito-suspension and β–sito–Alg/Ch/NPs were determined to be 28 ± 3%, and 64 ± 6%, respectively. The antioxidant activity of placebo Alg/Ch/NPs as control was also determined to be 16 ± 2%. The results indicated that the inhibition percentage of β–sito–Alg/Ch/NPs was more statistically significant than β–sito–suspension (*p* < 0.05). The high antioxidant activity was shown by β–sito–Alg/Ch/NPs plausibly due to the dissolved state of the encapsulated drug within the polymeric matrix, which protects the drug and provides stability against degradation and drug loss [58].

#### 5.6.10. Formulation Stability 

**Figure 13:**
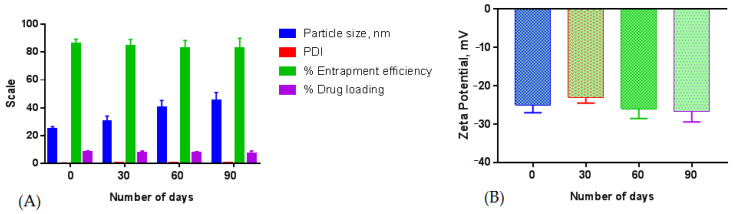
Stability assessment of β–sito–Alg/Ch/NPs for a period of three months at room temperature (25 ± 2 °C) shows alteration in particle size, PDI, % entrapment efficiency, and % drug loading (**A**); and zeta potential (**B**). Stability of the formulation illustrated that insignificant (*p* > 0.05) changes in the parameters under study at the designated condition.

The sample for the stability was assessed for a given time interval and the results recorded have shown changes in the particle size (nm), % entrapment efficiency, % drug loading, PDI, and zeta potential of the NPs, but these changes were statistically insignificant, clarifying that in-house build formulation was consistent and stable for the said period (*p* > 0.05). The particle size of the nanosystem goes on increasing while increasing the number of days during storage; this may be due to some degree of agglomeration among the particle surface resulting in increased size. The entrapment efficiency was also decreased due to hydration of aqueous medium into the NPs which may lead to the leaking of the drug from the polymeric matrix of the particles. The stability study data of β–sito–Alg/Ch/NPs are shown in Figure 13.

## 6. Conclusions

The in-house synthesized β–sito–Alg/Ch/NPs were successfully designed and characterized in vitro which showed enhanced drug release compared with a drug suspension. The presented study has shown the impact of selected variables chitosan (X1); sodium alginate (X2); and calcium chloride (X3) as per design-expert levels of low and high value on various responses; particle size (Y1), PDI (Y2); and % entrapment efficiency (Y3) of the NPs. The optimum formulation of the desirability value (0.851), with the composition of X1:X2:X3 (0.15%:0.50%:15.07 mM), observed a robust and consistent preparation. The Malvern Zetasizer indicated narrow particle-size distribution, and the TEM study analyzed the NPs were spherical in shape, uniform, de-aggregated and distributed finely in the preparation. The chitosan and alginate layer modulated drug release via diffusion from the NPs and demonstrated a controlled and prolonged release in PBS of pH 5.5 for a period of 96 h. The drug release mechanism from NPs indicated that Fickian diffusion with Korsmeyer–Peppas was the best-fitted model with the regression value of (R^2^ = 0.9715). Moreover, analytical studies of the optimized formulation further corroborated the stability of the alginate/chitosan complex and molecular state of the drug in the formulation. The everted sac permeability assessed higher intestinal permeation of drug from β–sito–Alg/Ch/NPs than β–sito–suspension. The cytotoxicity studies demonstrated that the β–sitosterol-loaded Alg/Ch/NPs had higher toxicity towards the MCF-7 cell line as compared to β–sito–suspension. The in vivo study pointed out ~3.41-times improved oral absorption of β–sitosterol from β–sito–Alg/Ch/NPs than drug suspension. Radical scavenging activity in preventing the generation of radicals by the formulation was more pronounced than β–sito–suspension. The stability study revealed that formulation β–sito–Alg/Ch/NPs remained stable for 3 months at room temperature. The outcomes of the current studies revealed that the developed β–sito–Alg/Ch/NPs formulation is promisingly useful in breast cancer treatment.

## Figures and Tables

**Table 1:** Different levels of factors and their responses as low, medium, and high in optimization of β–sito–Alg/Ch/NPs in Box Behnken design.

Factors	Levels Used		
	Low (−1)	Medium (0)	High (+1)
X1: Chitosan, %*w*/*v*	0.15	0.22	0.30
X2: Sodium alginate, %*w*/*v*	0.25	0.38	0.50
X3: Calcium chloride, mM	15	22.5	30
**Responses**			
Y1: Particle size, nm		Minimize	
Y2: PDI		Minimize	
Y3: Entrapment efficiency, %		Maximize	

**Table 2:** The results of in vitro assessed parameter of formulations of Run (1–17) for responses in optimization process of β–sito–Alg/Ch/NPs.

Runs	Independent Variables			Responses		
	X1, %*w*/*v*	X2, %*w*/*v*	X3, mM	Y1, nm	Y2	Y3, %
* 1	0.15	0.38	15.00	21.00	0.120	77.00
2	0.22	0.50	30.00	42.00	0.310	78.00
3	0.30	0.38	30.00	40.00	0.61	91.00
* 4	0.22	0.25	15.00	45.00	0.204	72.00
5	0.22	0.38	22.00	34.00	0.380	75.00
* 6	0.22	0.38	22.00	34.90	0.420	76.00
7	0.30	0.50	22.00	31.00	0.570	89.00
8	0.30	0.38	15.00	42.00	0.510	86.00
9	0.22	0.25	30.00	43.00	0.530	76.00
10	0.15	0.25	22.50	23.00	0.260	73.00
* 11	0.15	0.50	22.50	16.00	0.330	79.00
12	0.22	0.38	22.50	32.00	0.340	78.00
13	0.22	0.38	22.50	33.00	0.360	76.30
* 14	0.22	0.38	22.50	33.60	0.402	77.00
15	0.22	0.50	15.00	35.00	0.310	76.00
16	0.30	0.25	22.00	38.00	0.730	88.00
17	0.15	0.38	30.00	27.00	0.350	77.60

X1: Chitosan, %*w*/*v*, X2: Sodium alginate, %*w*/*v*, X3: Calcium chloride (mM), Y1: Particle size (nm), Y2: PDI, Y3: % Entrapment efficiency. * Replicas.

**Table 3:** The regression analysis results of responses in model summary statistics.

Model	R^2^	Adjusted R^2^	Predicted R^2^	SD	CV%	Desirability
Response: Y1						0.851
Quadratic	0.9857	0.9674	0.8510	1.41	4.21	
2FI	0.8012	0.6819	0.0006	4.39	-	
Linear	0.2448	0.0705	−0.5756	7.50	-	
Cubic						
Response: Y2						0.851
Quadratic	0.9772	0.9480	0.7845	0.036	9.96	
2FI	0.5503	0.2805	−1.2548	0.13	-	
Linear	0.1480	−0.0486	−0.7686	0.16	-	
Cubic	0.032	0.9897	0.9588	-	-	
Response: Y3						0.851
Quadratic	0.9884	0.9736	0.9545	0.93	1.17	
2FI	0.6350	0.4161	−0.5658	4.36	-	
Linear	0.6118	0.5223	0.2619	3.94	-	
Cubic						

**Table 4:** The optimum composition of formulation of both observed versus predicted responses of β–sito–Alg/Ch/NPs.

Independent Variables	Optimized Composition	Predicted Response			Observed Response			% Error
		Y1, nm	Y2 (%)	Y3 (%)	Y1, nm	Y2	Y3 (%)	
X1:X2:X3	0.15%:0.50%:15.07 mM	17.14	0.240	79.19	25 ± 1	0.231	86 ± 3	Y1 = 4.7 Y2 = 3.8 Y3 = 8.7

**Table 5:** Pharmacokinetic profile of β–sito–Alg/Ch/NPs and β–sito–suspension.

Formulation	AUC_0−t_ (μg × h/mL)	T_max_ (h)	C_max_	t_1/2_ (h)	k_e_ (h^−1^)
β–sito–Alg/Ch/NPs	1080 ± 1	4	180 ± 0.02	5 ± 0.1	0.2 ± 0.01
β–sito–suspension	317 ± 1	4	56 ± 0.2	2 ± 0.3	0.3 ± 0.02

## Data Availability

Not applicable.
